# *De Novo* Sequencing and Comparative Analysis of *Schima superba* Seedlings to Explore the Response to Drought Stress

**DOI:** 10.1371/journal.pone.0166975

**Published:** 2016-12-08

**Authors:** Bao-cai Han, Wei Wei, Xiang-cheng Mi, Ke-ping Ma

**Affiliations:** 1 State Key Laboratory of Vegetation and Environmental Change, Institute of Botany Chinese Academy of Sciences (IBCAS), Beijing, China; 2 University of Chinese Academy of Sciences, Beijing, China; Chinese Academy of Medical Sciences and Peking Union Medical College, CHINA

## Abstract

*Schima superba* is an important dominant species in subtropical evergreen broadleaved forests of China, and plays a vital role in community structure and dynamics. However, the survival rate of its seedlings in the field is low, and water shortage could be a factor that limits its regeneration. In order to better understand the response of its seedlings to drought stress on a functional genomics scale, RNA-seq technology was utilized in this study to perform a large-scale transcriptome sequencing of the *S*. *superba* seedlings under drought stress. More than 320 million clean reads were generated and 72218 unique transcripts were obtained through *de novo* assembly. These unigenes were further annotated by blasting with different public databases and a total of 53300 unique transcripts were annotated. A total of 31586 simple sequence repeat (SSR) loci were presented. Through gene expression profiling analysis between drought treatment and control, 11038 genes were found to be significantly enriched in drought-stressed seedlings. Based on these differentially expressed genes (DEGs), Gene Ontology (GO) terms enrichment and Kyoto Encyclopedia of Genes and Genomes pathway (KEGG) enrichment analysis indicated that drought stress caused a number of changes in the types of sugars, enzymes, secondary mechanisms, and light responses, and induced some potential physical protection mechanisms. In addition, the expression patterns of 18 transcripts induced by drought, as determined by quantitative real-time PCR, were consistent with their transcript abundance changes, as identified by RNA-seq. This transcriptome study provides a rapid method for understanding the response of *S*. *superba* seedlings to drought stress and provides a number of gene sequences available for further functional genomics studies.

## Introduction

*Schima superba*, belonging to the tea family (Theaceae) and distributed over large areas in subtropical China, is a dominant species in subtropical evergreen broadleaved forest [1]. It is also an important species in forest fire prevention, owing to its fire resistance [2]. As the community skeleton, the regeneration of its seedlings is a key process for forest community succession and ecological restoration of vegetation [3]. However, the wild population of *S*. *superba* survival rate is very low during the early growth stage. It was found that only 11% young seedlings could enter the next stage class [4]. Drought stress could play an important role in seedling survival and forest regeneration. Studies have indicated that even in tropical forest ecosystems characterized by high rainfall, drought can change community structure [5]. In Gutianshan (GTS) forest dynamics plot (FDP), there are two obviously dry seasons, from July to August and October to February [6]. Therefore, it is vital to study the response of forest species to drought stress, especially dominant species.

Controlled experiments are an effective method to understand the mechanisms of the interactions between plants and an aimed environmental factor in plant ecophysiology. However, only a few representative morphological and physiological traits can be selected as response indices, although higher-quality instruments are becoming more portable, capable, and automatic [7]. Therefore, it is hard to discern the comprehensive adaptive strategies of the plants. Moreover, with most of these indices, it is difficult to ensure synchronization of measurements among different samples because of the long time required to take measurements, which would influence the correlation among different samples and among the different replicates.

With the development of the next generation sequencing, RNA-seq enables us to study transcriptomes without the need for a whole genome sequence, and this is particularly attractive in research on non-model organisms [8]. This approach could offer both annotation with high resolution and quantifiable gene expression levels which could reflect the developing situation or the response to environmental stress at the functional genomics scale [9]. In addition, RNA-seq could drastically reduce the time and costs for obtaining sequence data and could guarantee sampling synchronicity among different samples and among different replicates. This technology, coupled with analytical approaches and molecular datasets, such as the Gene Ontology [10] and KEGG [11], may open up immense possibilities for large-scale sequencing of ecologically important species responding to their environments, which was unimaginable in earlier days.

In this study, we conducted large-scale transcriptome sequencing of *S*. *superba* under drought stress with Illumina HiSeq 2000 sequencing platform. We obtained a functional genomics resource of *S*. *superba* and finely identified stress-related transcripts through DEG analysis to aid in understanding the mechanisms of drought stress response. The candidate molecular makers, SSRs, were developed in order to facilitate further research, such as genotype–phenotype relationships in natural populations. Finally, 18 transcripts were selected for RT-PCR arrays, validating our study of the response to drought stress in *S*. *superba*.

## Results

### Sequencing and *de novo* assembly of *Schima superba* transcriptome

**Fig 1:**
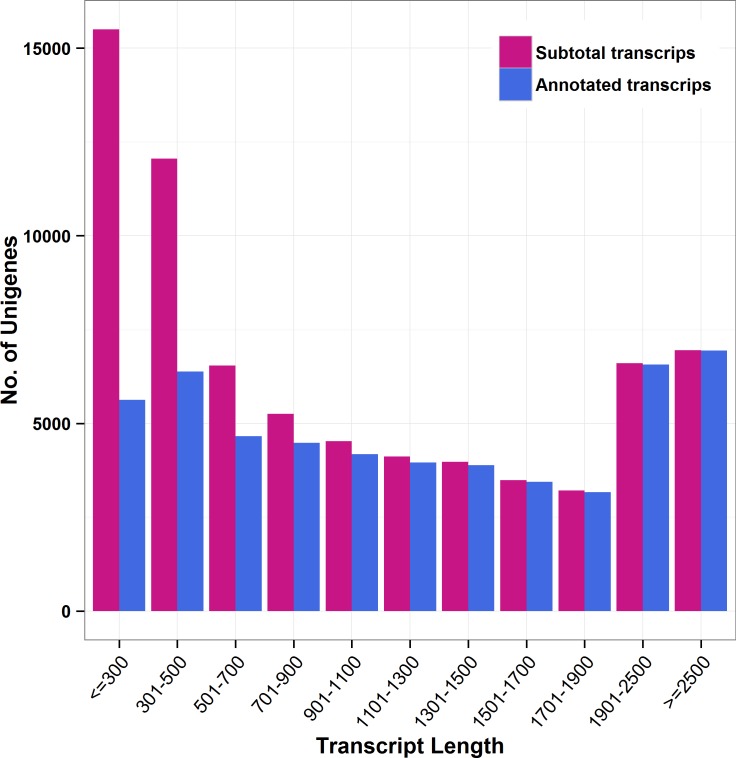
Length distribution of *Schima superba* unique transcripts.

More than 340 million raw reads were obtained from sequencing, and more than 320 million clean reads remained after trimming adaptors and low-quality reads. After *de novo* assembly, 72218 unigenes were obtained; average length was 1124 bp, and N50 was 1819 bp. The length distribution is shown in Fig 1. The Illumina HiSeq 2000 sequencing data of this species were deposited into the NCBI SRA database under the accession number SRX1531873.

### Annotation of *S*. *superba* unigenes

Different databases were blasted to annotate the assembled unigenes of *S*. *superba*, and 50488 (69.9%), 47678 (66.0%), 35040 (48.5%), 20708 (28.7%), and 30924 (42.8%) unigenes were annotated to the NR, NT, Swiss-Prot, KOG, and KEGG database with significant hits (E-Value < = le-5), respectively. In total, 53300 unigenes were annotated, and the distribution of the annotated unigenes is shown in Fig 1.

**Fig 2:**
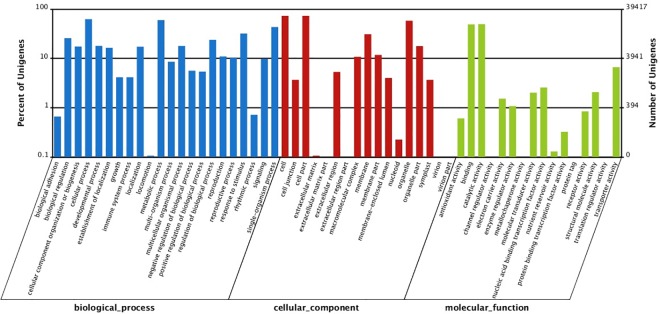
Gene ontology categorization of the assembled unigenes. The right Y-axis represents the number of genes in a category; the left Y-axis indicates the percentage of a specific category of genes in each main category.

The annotated unigenes were then assigned into GO terms. A total of 39417 unigenes were assigned with at least one GO term (Fig 2), among which 30237 (76.7%) were assigned in the biological process category, 29838 (75.7%) in the molecular function category, and 30605 (77.6%) in the cellular component category. 20297 (51.5%) unigenes were assigned in all three categories. In terms of biological process, ‘cellular process’ (24620), ‘metabolic process’ (23596) and ‘single-organism process’ (17065) were the top three GO terms. In terms of cellular component, the top three GO terms were related to the ‘cell’ (28800), ‘cell part’ (28799), and ‘organelle’ (22893). The analysis of molecular function showed that ‘catalytic activity’ (19525), ‘binding’ (19321), and ‘transporter activity’ (2614) were the most represented categories.

**Fig 3:**
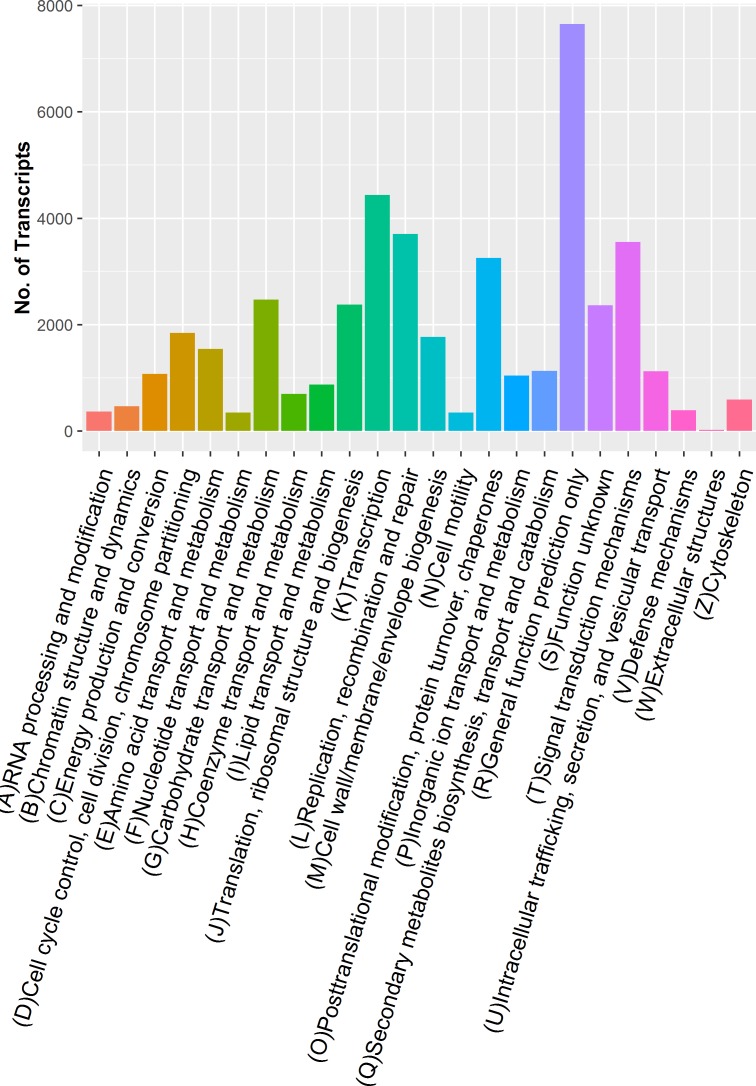
KOG classification of the putative proteins. The Y-axis indicates the number of unigenes in a specific functional cluster.

In order to classify the orthologous gene products, 20708 unigenes were subdivided into 25 classifications (Fig 3). In these classifications, the cluster of ‘general function prediction only’ (7649) represented the largest group, followed by ‘transcription’ (4439) and ‘replication, recombination and repair’ (3704). Only a few unigenes were aligned to ‘nuclear structure’ (12) and ‘extracellular structures’ (23).

The pathways were analyzed using the KEGG annotation system. In all, 128 pathways were identified. Among them, ‘metabolic pathways’ (6692) and ‘biosynthesis of secondary metabolites’ (3111) possessed the largest number of unigenes, whereas ‘tropane’ (4) and ‘valine’ (3) pathways had the least. The KEGG pathways are shown in S1 Table.

In total, 11038 significant DEGs were identified between drought treatment and the control, among which 6498 were up-regulated and 4540 down-regulated. It is necessary to understand the functional distribution of *S*. *superba* response to drought stress based on DEGs. In our present study, the GO enrichment of DEGs is shown in S2 and S3 Tables. In the biological process category, the top three enriched terms of up-regulated transcripts were ‘photosynthesis, light harvesting’, ‘transmembrane receptor protein tyrosine kinase signaling pathway’, and ‘response to red light’; the top three enriched terms of down-regulated transcripts were ‘cysteine biosynthetic process’, ‘glucosinolate biosynthetic process’, and ‘asymmetric cell division’. The enriched term of up-regulated transcripts ‘response to water deprivation’ further demonstrates the reliability of our drought treatment. In the cell components category, ‘chloroplast thylakoid membrane’, ‘extracellular region’, and ‘apoplast’ were the three dominant enriched terms of up-regulated transcripts; ‘anchored to plasma membrane’, ‘cell wall’, and ‘thylakoid’ were the three dominant enriched terms of down-regulated transcripts. For the molecular function category, ‘inositol 3-alpha-galactosyltransferase activity’, ‘NADP binding’, and ‘NADPH dehydrogenase activity’ were the mostly highly enriched terms of up-regulated transcripts; ‘identical protein binding’, ‘cation binding’, and ‘oxidoreductase activity, acting on NAD(P)H, quinone or similar compound as acceptor’ were the mostly highly enriched terms of down-regulated transcripts.

**Fig 4:**
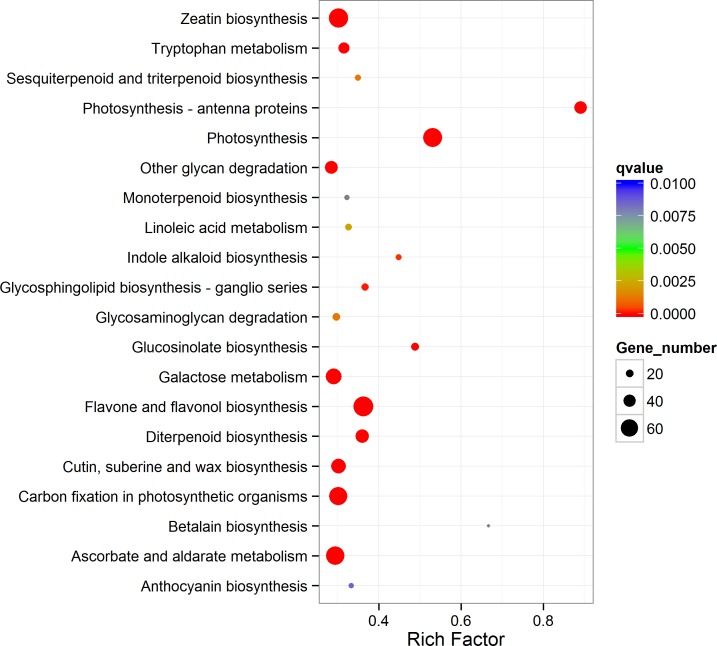
Enriched KEGG of up-regulated transcripts of the annotated DEGs. The left Y-axis indicates the KEGG pathway. The X-axis indicates the Rich factor. A high q value is represented by blue, and a low q value is represented by red. “Rich factor” in Fig 4 represents the number of up-regulated DEGs in the related pathway divided by the number of all the annotated genes in this pathway. “qvalue” is the name of the adjusted p-values obtained using an optimised False Discovery Rate (FDR) approach.

The enriched KEGG pathways are shown in S4 and S5 Tables. There were 35 enriched pathways of up-regulated transcripts and 23 enriched pathways of down-regulated transcripts. ‘Photosynthesis—antenna proteins’, ‘photosynthesis’, and ‘metabolic pathways’ were the top enriched pathways of up-regulated transcripts, whereas ‘spliceosome’, ‘RNA transport’, and ‘protein processing in endoplasmic reticulum’ were the top three enriched pathways of down regulated transcripts. The enriched pathways of up-regulated transcripts are shown in Fig 4.

### The correlation among the replicates and qRT-PCR analysis

**Fig 5:**
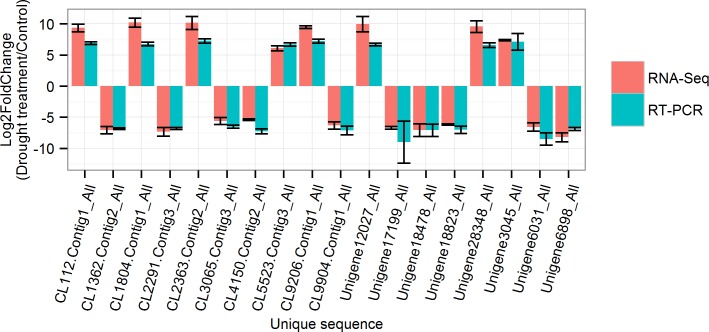
Verification of 18 putative genes that are involved in the drought response by qRT-PCR. The left Y-axis indicates the normalized expression pattern of the DEGs under drought. Error bars represent ±1 SE.

The high correlation of expression profiles among biological replicates indicated the robustness of our RNA-seq dataset (correlation coefficients between DT1 and DT2, DT1 and DT3, DT2 and DT3, CK1 and CK2, CK1 and CK3, CK2 and CK3 were 0.8873, 0.8410, 0.9564, 0.8308, 0.8124 and 0.9203, respectively, Table 1). Meanwhile, qRT-PCR assays of 18 selected drought-responsive unique transcripts were conducted. The results indicate that although there is some variation between the RNA-seq expression and the qRT-PCR analyses (Fig 5), there was still enough consistency to verify the two analysis methods.

**Table 1:** Correlation coefficient of transcriptome profiles among RNA-seq samples.

	T1	T2	T3	CK1	CK2	CK3
**T1**	1	0.8873	0.9564	0.4586	0.5057	0.4654
**T2**	0.8873	1	0.8410	0.5035	0.5525	0.5010
**T3**	0.9564	0.8410	1	0.4580	0.5080	0.4637
**CK1**	0.4586	0.5035	0.4580	1	0.8308	0.8124
**CK2**	0.5057	0.5525	0.5080	0.8308	1	0.9203
**CK3**	0.4654	0.5010	0.4627	0.8124	0.9203	1

DT1, DT2, DT3: drought treated samples; CK1, CK2, CK3: control samples.

### The identification of SSRs

In this study, a total of 31586 distinct SSR loci were identified from di- to hexa-nucleotide. Dinucleotides were the most commonly identified loci and quad nucleotides were the least common, with 17325 and 479, respectively. The AG/CT was the most frequent dinucleotide SSR repeat, accounting for 13806 loci, and the AAG/CTT was the most frequent trinucleotide SSR and accounted for 1487 loci (Table 2).

**Table 2:** Summary of EST-SSR detected from transcriptome data.

Motif length	Repeat numbers								Total (%)
	4	5	6	7	8	9	10	>10	
**Di-nucleotide**	-	-	3224	2509	3194	4601	3094	703	17325
**Tri-nucleotide**	-	3309	1684	1085	166	12	-	-	6262
**Quad-nucleotide**	-	387	86	4	1	1	-	-	479
**Penta-nucleotide**	691	86	3	-	-	-	-	-	780
**Hexa-nucleotide**	886	12	-	1	1	-	-	-	900
**Total**	1577	3794	4997	3599	3362	4614	3094	709	25746

## Discussion and Conclusion

### *Schima superba* functional genomics characteristics

*S*. *superba* is an important dominant species in subtropical China. Our work generated approximately 33 Gb of sequence data and more than 320 million clean sequence reads, which guarantees the accuracy and reliability for the following analysis. In the final assembly, we obtained 72218 transcripts, with an N50 of 1819. 53300 transcripts were ultimately annotated. To our knowledge, this is the first *S*. *superba* functional genomics database produced based on the Illumina HiSeq 2000 platform.

Molecular markers are useful for further research in breeding or other molecular studies [12–14]. In this study, we found a large number of polymorphisms within the transcriptome (Table 2), among which, the number of dinucleotide SSRs (67.3%) was the largest. AG/CT and AAG/CTT were the most frequent dinucleotide and trinucleotide SSRs. Similarly, these two types SSRs were also the most frequent di- and tri-nucleotide SSRs identified in *Sophora moorcroftiana* [15], *Heveabra siliensis* [16], *Capsicum annuum* [17], and *Sesamum indicum*[18], which belong to different orders in the higher plant kingdom. This indicates that these two types SSRs may be somewhat evolutionary conserved in the higher plant kingdom, which is interesting and points to the need for further research.

### Strategies of *S*. *superba* seedlings response to drought stress

In this study, there was a high correlation among the biological replicates (Table 1), and the comparison between the qRT-PCR assays of 18 selected drought-responsive unique transcripts and their RNA-Seq profile (Fig 5) assured us in the reliability of the drought treatment and RNA-seq data. Through the DESeq analysis, we found 11038 DEGs. GO and KEGG enrichment analysis revealed that the differentially expressed genes were involved in important GO terms and pathways, including the enriched biological process GO term of up-regulated transcripts ‘response to water deprivation’, confirming the effectiveness of our drought treatment.

When plants encounter water stress, the decreased cellular volume causes crowding of cytoplasmic components. Then the increasingly viscous cell contents would increase the chance for molecular interactions that can cause protein denaturation and membrane fusion [19–20]. A large number of compounds are reported to aid in avoiding these adverse interactions by maintaining the membrane and stabilizing the structure of proteins, such as proline, flavonoids, glycine-betaine, trehalose, sucrose, and oligosaccharides [21]. The enriched terms of up-regulated transcripts of flavonoids, trehalose, sucrose, and oligosaccharides were observed in this study. Trehalose are reported to efficiently stabilize dehydrated enzymes, proteins, and lipid membranes but not to regulate water potential [22–23]; whereas galactinol and raffinose also function as osmoprotectants in drought stress tolerance [24]. These compounds would play an important function in stabilizing the membrane and the structure of proteins in the response of *S*. *superba* seedlings to drought.

Stomatal limitation would also reduce photosynthesis under drought stress, produce ROS and may finally cause the destruction of the structure of photosystem [25–26]. Many mechanisms are developed to avoid the destruction of the structure of photosystem. For example, photosystem II oxygen-evolving protein is essential for efficient and stable oxygen evolution. Even a lower level of oxygen- evolving activity could limit the photosynthetic activity so as to avoid the production of the ROS [27], and ferredoxin—NADP+ reductase (FNR), participating in the NADE'+ photoreduction and nitrate assimilation pathways, involve in free radical scavenging to reduce oxidative challenges [28]. In our study, transcripts encoding FNR and encoding photosystem II oxygen-evolving enhancer protein are accounting for the vast majority in photosynthesis pathway. The significant up-regulation of them indicates that the seedlings were preventing the structure of photosystem from destructing.

Besides the absorption of light and the transfer of the excitation energy to the photochemical reaction centers, chlorophyll a/b proteins (CAB) in the light-harvesting complex (LHC) of photosystem II play a vital role in protecting against photoinhibition by nonphotochemical quenching [29–30]. In our study, all 29 transcripts encoding CAB proteins were up-regulated in ‘Photosynthesis—antenna proteins’, which suggests that drought stress has triggered the protection against intense light by nonphotochemical quenching. Imbalances of photosynthesis are often coupled to increased production of reactive oxygen species (ROS) and ultimately results in oxidative stress. Plants have developed very efficient enzymatic antioxidant defense systems, such as superoxide dismutase, SOD; catalase, CAT; ascorbate peroxidase, APX; glutathione reductase, GR; monodehydroascorbate reductase, MDHAR; dehydroascorbate reductase, DHAR; glutathione peroxidase, GPX, to defend against oxidative stress damages by scavenging ROS [31]. In our study, all three transcripts encoding GR and the unique transcript encoding APX were down-regulated, whereas the unique transcript encoding DHAR, the unique transcript encoding MDHAR, and two transcripts encoding SOD were up-regulated. These results suggest the seedlings have activated mechanisms to scavenge ROS, and there may be different regulatory mechanisms between the different enzymatic pathways, forming a complex regulatory network.

Flavonoids are known to be not only functional in UV photoprotection, but also scavenge ROS, maintain the cell osmotic pressure, and act as the signal of environmental stress [32]. Leucoanthocyanidin reductase (LAR) and anthocyanidin synthase (ANS) are important in biosynthetic and metabolic pathways. LAR is proposed to convert flavan-3,4-diols (leucoanthocyanidins) to 2,3-trans-flavan-3-ols, which is a “starter unit” for tannin condensation [33]. ANS plays an important role in the biosynthesis of anthocyanins, and competes with LAR [34]. In the enriched of up-regulated transcripts ‘flavonoid biosynthesis’ pathway, we found that the two transcripts encoding ANS were up-regulated, but the unique transcript encoding LAR was down-regulated. This suggests that ANSs are active during water stress and anthocyanins are being synthesized, which is consistent with morphological observations. That is, when the *S*. *superba* seedlings encounter water stress, the color of the leaves changes.

9-*cis*-epoxycarotenoid dioxygenase (NCED) and zeaxanthin epoxidase (ZEP) are known to be important for ABA biosynthesis [35]. ABA biosynthesis may be regulated by the amount of *NCED* mRNA in the leaf, whereas *ZEP* mRNA has a positive drought response in roots but not in leaves, as reported in *Nicotiana plumbaginifolia* [36] and *Lycopersicon esculentum* [37]. In our study, we found that the five transcripts encoding NCED were down-regulated but one was up-regulated. However, two transcripts encoding ZEP were up-regulated but two others were down-regulated. These results are different from those of previous study [36–37], suggesting that ZEP and NCED perform different regulatory functions from those described in the previous studies.

A cuticle layer covers the surface of higher plants and forms the contact zone between the plant and the environment [38]. Cuticular waxes and cutin monomers are the major components of the plant cuticle, and they play an important role in protecting aerial organs from damage caused by drought and other environmental stresses [39–40]. The enriched pathway of up-regulated transcripts of ‘cutin, suberine, and wax biosynthesis’ includes these products, which serve as protective mechanisms in response of the *S*. *superba* seedlings to drought stress. This discovery demonstrates the advantage of transcriptomics, because traditional physiological methods would be unable to detect the responses of this potential physical protection mechanism in such a short time.

In summary, RNA-seq combined with DEGs analysis provided comprehensive information on the functional genomics and gene expression of *S*. *superba* seedling response to drought stress. Based on the enrichment of KEGG pathways and GO analysis, we found that the differentially expressed genes were involved in important pathways and GO terms, which are reported to play important roles in preventing *S*. *superba* seedlings from drought stress. These large-scale transcriptomic data and DEGs give us a full picture of the regulatory process, which could not be reached by traditional physiological measuring. For example, it is difficult, if even possible, to detect the potential physical protection of wax and cutin biosynthesis in traditional methods during such a short treatment time. This study may provide a new insight for further study in ecology. In the future, more sampling points in different treatment times may reveal higher resolution gene or metabolite responses, which could indicate the stress degree, and be applied to ecophysiology studies and other related disciplines.

## Materials and Methods

### Ethics Statement

This study was approved by the Gutianshan Research Station of Forest Biodiversity and Climate Change, IBCAS and Gutianshan National Nature Reserve Authority. *Schima superba* is not an endangered or protected species.

### Plant material and drought stress treatments

One-year-old *S*. *superba* seedlings were obtained from Dexing nursery, Dexing City, Jiangxi Provence in August 2013 and cultivated in pots (15.5cm in upper diameter, 10cm in lower diameter, and 11cm high) filled with the soil gained from the original distribution of *S*. *superba* in GTS. The seedlings were well watered and incubated for 9 weeks in three plant growth chambers under 28°C, 50% humidity during the day for 16 hours (continuous light 200 μmolm^-2^s^-1^) and 22°C, 72% during the night for 8 hours.Then 36 seedlings with the similar growing consistency (about 35 cm high) were selected and randomly assigned into two groups: control sample (CK) and drought-treatment (DT).Each group was performed with three independent biological replicates (CK1, CK2, CK3, DT1, DT2, DT3). Thus, there were 6 seedlings included in each replicate of each group. Drought stress causes stomatal closure and induces the expression of drought stress-related genes [41]; we partly followed the sampling method of Raj et al. (2011) [42] to monitor when the plants reached the state of drought stress. Specifically, the stomatal conductance of the first fully expanded leaves was measured every day in the midday with IR gas analyzer (LI-6400XT Portable Photosynthesis System; LI-COR Biosciences). Significant differences in stomatal conductance over two consecutive days between the drought treatment and the control indicated the response to drought stress. In this study, the stomatal conductance was significantly different (Wilcoxon test, p<0.05) at the 5^th^and 6^th^day, and at the 7^th^day the first fully expanded leaves of six individuals were harvested from each replicate of each group (the stomatal conductance data is shown in S6). Samples were quickly frozen in liquid nitrogen for further laboratory studies.

### Total RNA extraction, RNA-seq library construction, and sequencing

The first fully expanded leaves of 6 seedlings, cultivated in each replicate of each group, were pooled as one sample. So finally there were a total of 6 samples used for sequencing.

Total RNA was extracted using RNeasy Plant Mini Kit, followed by RNA purification with the RNeasy Mini Elute Cleanup Kit (Qiagen, Germany), according to the manufacturer’s instruction. RNA was quantified using the Agilent 2100 Bioanalyzer. The purity of all RNA samples was assessed at an absorbance ratio of OD260/280 and OD260/230.

For mRNA library construction, RNA samples were prepared using the TruSeq RNA Sample Preparation Kit according to the manufacturer’s protocol. Each of these six libraries was sequenced using Illumina HiSeq 2000 platform. Each sequenced sample yielded 2 × 90-bp independent reads.

### Data processing, *de novo* assembly, and functional annotation

The raw reads were purified by trimming the adapter sequences and low quality sequences (reads with N percentage over 5% and those containing more than 20% nucleotides Q-value < 20) on both ends. After preprocessing, we obtained quality filtered short reads of ~ 5.53 Gb, ~5.13 Gb, and ~ 5.22 Gb in drought treatment and ~ 5.43 Gb, ~ 5.26 Gb, and ~ 5.53 Gb in the control. The final clean reads were assembled using the Trinity software with default parameters [43]. After that, the transcripts of six samples were further spliced and assembled to obtain non-redundant unigenes by TGICL with the minimum overlap length of 100 bp [44]. These final unigenes were used for further analysis in this study.

Unigene sequences were aligned to protein databases of NR, Swiss-Prot, KEGG, and KOG (e-value<0.00001) by blastx and the nucleotide database NT (e-value<0.00001) by blastn. We decided the unigene sequence directions based on the best alignment results over all these databases. When different databases produced conflicting results, the sequence direction was assigned based on the following priority order: NR, Swiss-Prot, KEGG, and KOG. With NR annotation, GO annotation of unigenes was obtained using the Blast2GO program.

### Gene expression quantification, differential expression analysis and SSR detection

The clean short reads were mapped back onto the assembled transcriptome to gain the read count of each gene. DEGs (|log2foldchanges| > 1 and adjusted p-value < 0.01) between control and drought stress conditions were identified using the DESeq package [45] in R software. GO terms enrichment based on DEGs were identified using the GOseq package [46] in R software, and KEGG pathway enrichment analysis based on DEGs was performed using KOBAS [47]. SSRs of transcriptome were detected with MIcroSAtellite software (http://pgrc.ipk-gatersleben.de/misa/misa.html).

### Quantitative RT-PCR analysis

To validate the reliability of RNA-seq, 18 transcripts were selected for qRT-PCR validation. With DNase I treated RNA (2μg) of each sample, real-time PCR was performed under the following parameters: 95°C for 2 min, 40 cycles at 94°C for 10 s, 59°C for 10 s, and 72°C for 40 s in ABI ViiA 7 Real Time PCR System (Applied Biosystems). Each reaction was performed in triplicate. Melting curve analysis was used to verify the products. The target transcript copies were normalized by the reference 18S gene using the comparative ΔΔCT method [48]. All data are shown as the mean ± 1 SE and all primer information is provided in S7 Table.

## Supporting Information

S1 Table List of annotated KEGG pathways of *Schima superba* seedlings. (DOCX)

S2 Table GO enrichment of up-regulated DEGs in the drought stress treatment. (DOCX)

S3 Table GO enrichment of down-regulated DEGs in the drought stress treatment. (DOCX)

S4 Table KEGG pathway enrichment of up-regulated DEGs in the drought treatment. (DOCX)

S5 Table KEGG pathway enrichment of down-regulated DEGs in the drought treatment. (DOCX)

S6 Table The stomatal conductance (GS) data measured from the first six days (unit: mmol•m-2•s-1) (DOCX)

S7 Table Primers for qRT-PCR analysis. (DOCX)
