# Supplementary material for: De Novo Sequencing and Comparative Analysis of Schima superba Seedlings to Explore the Response to Drought Stress
Source: PLoS One. 2016 Dec 8;11(12):e0166975. doi: 10.1371/journal.pone.0166975 (PMC5145176; doi:10.1371/journal.pone.0166975)
Supplement: S1 Table — (DOCX) [file pone.0166975.s001.docx]

**S1 Table. List of annotated KEGG pathways of *Schima superba* seedlings.**

| **NO.** | **Pathway** | **Unigenes NO.** | **Pathway ID** | **Level** |
| --- | --- | --- | --- | --- |
| 1 | Endocytosis | 1231 | ko04144 | Cellular Processes |
| 2 | Phagosome | 313 | ko04145 | Cellular Processes |
| 3 | Peroxisome | 236 | ko04146 | Cellular Processes |
| 4 | Regulation of autophagy | 228 | ko04140 | Cellular Processes |
| 5 | Plant hormone signal transduction | 2041 | ko04075 | Environmental Information Processing |
| 6 | ABC transporters | 368 | ko02010 | Environmental Information Processing |
| 7 | Phosphatidylinositol signaling system | 270 | ko04070 | Environmental Information Processing |
| 8 | Spliceosome | 1201 | ko03040 | Genetic Information Processing |
| 9 | RNA transport | 1167 | ko03013 | Genetic Information Processing |
| 10 | Protein processing in endoplasmic reticulum | 864 | ko04141 | Genetic Information Processing |
| 11 | mRNA surveillance pathway | 686 | ko03015 | Genetic Information Processing |
| 12 | Ubiquitin mediated proteolysis | 624 | ko04120 | Genetic Information Processing |
| 13 | Ribosome | 579 | ko03010 | Genetic Information Processing |
| 14 | RNA degradation | 573 | ko03018 | Genetic Information Processing |
| 15 | Ribosome biogenesis in eukaryotes | 527 | ko03008 | Genetic Information Processing |
| 16 | RNA polymerase | 469 | ko03020 | Genetic Information Processing |
| 17 | Nucleotide excision repair | 225 | ko03420 | Genetic Information Processing |
| 18 | Basal transcription factors | 218 | ko03022 | Genetic Information Processing |
| 19 | Aminoacyl-tRNA biosynthesis | 193 | ko00970 | Genetic Information Processing |
| 20 | Base excision repair | 173 | ko03410 | Genetic Information Processing |
| 21 | DNA replication | 150 | ko03030 | Genetic Information Processing |
| 22 | Homologous recombination | 133 | ko03440 | Genetic Information Processing |
| 23 | Proteasome | 130 | ko03050 | Genetic Information Processing |
| 24 | SNARE interactions in vesicular transport | 123 | ko04130 | Genetic Information Processing |
| 25 | Protein export | 117 | ko03060 | Genetic Information Processing |
| 26 | Mismatch repair | 114 | ko03430 | Genetic Information Processing |
| 27 | Non-homologous end-joining | 28 | ko03450 | Genetic Information Processing |
| 28 | Sulfur relay system | 26 | ko04122 | Genetic Information Processing |
| 29 | Metabolic pathways | 6692 | ko01100 | Metabolism |
| 30 | Biosynthesis of secondary metabolites | 3111 | ko01110 | Metabolism |
| 31 | Glycerophospholipid metabolism | 1100 | ko00564 | Metabolism |
| 32 | Ether lipid metabolism | 894 | ko00565 | Metabolism |
| 33 | Purine metabolism | 798 | ko00230 | Metabolism |
| 34 | Starch and sucrose metabolism | 724 | ko00500 | Metabolism |
| 35 | Pyrimidine metabolism | 718 | ko00240 | Metabolism |
| 36 | Phenylpropanoid biosynthesis | 518 | ko00940 | Metabolism |
| 37 | Oxidative phosphorylation | 408 | ko00190 | Metabolism |
| 38 | Pentose and glucuronate interconversions | 360 | ko00040 | Metabolism |
| 39 | Flavonoid biosynthesis | 344 | ko00941 | Metabolism |
| 40 | Glycolysis / Gluconeogenesis | 337 | ko00010 | Metabolism |
| 41 | Amino sugar and nucleotide sugar metabolism | 330 | ko00520 | Metabolism |
| 42 | Stilbenoid, diarylheptanoid and gingerol biosynthesis | 290 | ko00945 | Metabolism |
| 43 | Cysteine and methionine metabolism | 252 | ko00270 | Metabolism |
| 44 | Pyruvate metabolism | 242 | ko00620 | Metabolism |
| 45 | Phenylalanine metabolism | 236 | ko00360 | Metabolism |
| 46 | Inositol phosphate metabolism | 228 | ko00562 | Metabolism |
| 47 | Glutathione metabolism | 226 | ko00480 | Metabolism |
| 48 | Zeatin biosynthesis | 225 | ko00908 | Metabolism |
| 49 | Ascorbate and aldarate metabolism | 217 | ko00053 | Metabolism |
| 50 | Carbon fixation in photosynthetic organisms | 209 | ko00710 | Metabolism |
| 51 | Limonene and pinene degradation | 208 | ko00903 | Metabolism |
| 52 | Glycine, serine and threonine metabolism | 202 | ko00260 | Metabolism |
| 53 | Cyanoamino acid metabolism | 196 | ko00460 | Metabolism |
| 54 | Flavone and flavonol biosynthesis | 193 | ko00944 | Metabolism |
| 55 | Galactose metabolism | 186 | ko00052 | Metabolism |
| 56 | Glyoxylate and dicarboxylate metabolism | 184 | ko00630 | Metabolism |
| 57 | Carotenoid biosynthesis | 177 | ko00906 | Metabolism |
| 58 | Arginine and proline metabolism | 176 | ko00330 | Metabolism |
| 59 | Terpenoid backbone biosynthesis | 175 | ko00900 | Metabolism |
| 60 | Glycosylphosphatidylinositol(GPI)-anchor biosynthesis | 164 | ko00563 | Metabolism |
| 61 | Cutin, suberine and wax biosynthesis | 162 | ko00073 | Metabolism |
| 62 | Tyrosine metabolism | 158 | ko00350 | Metabolism |
| 63 | Pentose phosphate pathway | 156 | ko00030 | Metabolism |
| 64 | N-Glycan biosynthesis | 152 | ko00510 | Metabolism |
| 65 | Other glycan degradation | 151 | ko00511 | Metabolism |
| 66 | Glycerolipid metabolism | 145 | ko00561 | Metabolism |
| 67 | Fructose and mannose metabolism | 142 | ko00051 | Metabolism |
| 68 | Citrate cycle (TCA cycle) | 139 | ko00020 | Metabolism |
| 69 | alpha-Linolenic acid metabolism | 136 | ko00592 | Metabolism |
| 70 | Fatty acid metabolism | 136 | ko00071 | Metabolism |
| 71 | Phenylalanine, tyrosine and tryptophan biosynthesis | 136 | ko00400 | Metabolism |
| 72 | Nitrogen metabolism | 133 | ko00910 | Metabolism |
| 73 | Photosynthesis | 126 | ko00195 | Metabolism |
| 74 | Diterpenoid biosynthesis | 125 | ko00904 | Metabolism |
| 75 | Porphyrin and chlorophyll metabolism | 121 | ko00860 | Metabolism |
| 76 | Valine, leucine and isoleucine degradation | 121 | ko00280 | Metabolism |
| 77 | Alanine, aspartate and glutamate metabolism | 119 | ko00250 | Metabolism |
| 78 | Tryptophan metabolism | 111 | ko00380 | Metabolism |
| 79 | Lysine degradation | 110 | ko00310 | Metabolism |
| 80 | Propanoate metabolism | 110 | ko00640 | Metabolism |
| 81 | Ubiquinone and other terpenoid-quinone biosynthesis | 102 | ko00130 | Metabolism |
| 82 | Sphingolipid metabolism | 101 | ko00600 | Metabolism |
| 83 | Fatty acid biosynthesis | 100 | ko00061 | Metabolism |
| 84 | Steroid biosynthesis | 100 | ko00100 | Metabolism |
| 85 | beta-Alanine metabolism | 95 | ko00410 | Metabolism |
| 86 | Sulfur metabolism | 81 | ko00920 | Metabolism |
| 87 | Isoflavonoid biosynthesis | 78 | ko00943 | Metabolism |
| 88 | Pantothenate and CoA biosynthesis | 75 | ko00770 | Metabolism |
| 89 | Biosynthesis of unsaturated fatty acids | 75 | ko01040 | Metabolism |
| 90 | Butanoate metabolism | 74 | ko00650 | Metabolism |
| 91 | Isoquinoline alkaloid biosynthesis | 72 | ko00950 | Metabolism |
| 92 | Tropane, piperidine and pyridine alkaloid biosynthesis | 71 | ko00960 | Metabolism |
| 93 | Brassinosteroid biosynthesis | 71 | ko00905 | Metabolism |
| 94 | Valine, leucine and isoleucine biosynthesis | 70 | ko00290 | Metabolism |
| 95 | Histidine metabolism | 65 | ko00340 | Metabolism |
| 96 | Riboflavin metabolism | 65 | ko00740 | Metabolism |
| 97 | Glycosaminoglycan degradation | 64 | ko00531 | Metabolism |
| 98 | Fatty acid elongation | 63 | ko00062 | Metabolism |
| 99 | One carbon pool by folate | 63 | ko00670 | Metabolism |
| 100 | Benzoxazinoid biosynthesis | 59 | ko00402 | Metabolism |
| 101 | Selenocompound metabolism | 50 | ko00450 | Metabolism |
| 102 | Other types of O-glycan biosynthesis | 50 | ko00514 | Metabolism |
| 103 | Glycosphingolipid biosynthesis - ganglio series | 49 | ko00604 | Metabolism |
| 104 | Linoleic acid metabolism | 49 | ko00591 | Metabolism |
| 105 | Photosynthesis - antenna proteins | 46 | ko00196 | Metabolism |
| 106 | Glucosinolate biosynthesis | 43 | ko00966 | Metabolism |
| 107 | Lysine biosynthesis | 43 | ko00300 | Metabolism |
| 108 | Vitamin B6 metabolism | 43 | ko00750 | Metabolism |
| 109 | Sesquiterpenoid and triterpenoid biosynthesis | 40 | ko00909 | Metabolism |
| 110 | Nicotinate and nicotinamide metabolism | 39 | ko00760 | Metabolism |
| 111 | Arachidonic acid metabolism | 31 | ko00590 | Metabolism |
| 112 | Monoterpenoid biosynthesis | 31 | ko00902 | Metabolism |
| 113 | Anthocyanin biosynthesis | 30 | ko00942 | Metabolism |
| 114 | Thiamine metabolism | 29 | ko00730 | Metabolism |
| 115 | Indole alkaloid biosynthesis | 29 | ko00901 | Metabolism |
| 116 | Folate biosynthesis | 28 | ko00790 | Metabolism |
| 117 | Synthesis and degradation of ketone bodies | 23 | ko00072 | Metabolism |
| 118 | Glycosphingolipid biosynthesis - globo series | 22 | ko00603 | Metabolism |
| 119 | Taurine and hypotaurine metabolism | 19 | ko00430 | Metabolism |
| 120 | C5-Branched dibasic acid metabolism | 12 | ko00660 | Metabolism |
| 121 | Lipoic acid metabolism | 10 | ko00785 | Metabolism |
| 122 | Caffeine metabolism | 5 | ko00232 | Metabolism |
| 123 | Biotin metabolism | 4 | ko00780 | Metabolism |
| 124 | Betalain biosynthesis | 3 | ko00965 | Metabolism |
| 125 | Plant-pathogen interaction | 2433 | ko04626 | Organismal Systems |
| 126 | Circadian rhythm - plant | 374 | ko04712 | Organismal Systems |
| 127 | Natural killer cell mediated cytotoxicity | 124 | ko04650 | Organismal Systems |
| 128 | Circadian rhythm - mammal | 96 | ko04710 | Organismal Systems |
