# Supplementary material for: De Novo Sequencing and Comparative Analysis of Schima superba Seedlings to Explore the Response to Drought Stress
Source: PLoS One. 2016 Dec 8;11(12):e0166975. doi: 10.1371/journal.pone.0166975 (PMC5145176; doi:10.1371/journal.pone.0166975)
Supplement: S2 Table — (DOCX) [file pone.0166975.s002.docx]

**S2 Table. GO enrichment of up-regulated DEGs in the drought stress treatment.**

| **GOID** | **Ontology** | **Term** | **P value** |
| --- | --- | --- | --- |
| GO:0000023 | BP | photosynthesis, light harvesting | 2.24E-31 |
| GO:0000038 | BP | transmembrane receptor protein tyrosine kinase signaling pathway | 2.04E-27 |
| GO:0000079 | BP | response to red light | 6.71E-27 |
| GO:0000096 | BP | oxidation-reduction process | 1.91E-24 |
| GO:0000226 | BP | regulation of meristem growth | 3.39E-24 |
| GO:0000271 | BP | photosynthesis | 3.45E-24 |
| GO:0000272 | BP | response to blue light | 1.81E-23 |
| GO:0000293 | BP | pentose-phosphate shunt | 3.10E-22 |
| GO:0000786 | BP | negative regulation of catalytic activity | 7.85E-20 |
| GO:0000911 | BP | regulation of cell size | 1.25E-19 |
| GO:0002679 | BP | anthocyanin accumulation in tissues in response to UV light | 5.94E-19 |
| GO:0003777 | BP | photosystem II assembly | 1.80E-18 |
| GO:0003855 | BP | chlorophyll biosynthetic process | 7.25E-18 |
| GO:0003959 | BP | stomatal complex morphogenesis | 2.04E-17 |
| GO:0004021 | BP | response to far red light | 4.81E-17 |
| GO:0004047 | BP | plant-type cell wall organization | 6.35E-17 |
| GO:0004170 | BP | isopentenyl diphosphate biosynthetic process, mevalonate-independent pathway | 1.38E-16 |
| GO:0004180 | BP | polysaccharide biosynthetic process | 3.38E-16 |
| GO:0004252 | BP | plant-type cell wall biogenesis | 6.84E-16 |
| GO:0004375 | BP | cell tip growth | 3.52E-15 |
| GO:0004553 | BP | carbohydrate metabolic process | 2.71E-14 |
| GO:0004565 | BP | plastid organization | 6.50E-14 |
| GO:0004568 | BP | photosynthesis, light reaction | 1.80E-13 |
| GO:0004650 | BP | starch biosynthetic process | 3.27E-13 |
| GO:0004764 | BP | positive regulation of catalytic activity | 3.72E-12 |
| GO:0004857 | BP | microtubule-based movement | 6.16E-12 |
| GO:0005089 | BP | photosynthetic electron transport in photosystem I | 7.41E-12 |
| GO:0005242 | BP | regulation of hormone levels | 2.08E-11 |
| GO:0005506 | BP | rRNA processing | 2.29E-11 |
| GO:0005576 | BP | response to karrikin | 3.05E-11 |
| GO:0005618 | BP | polysaccharide catabolic process | 3.16E-11 |
| GO:0005874 | BP | maltose metabolic process | 4.92E-11 |
| GO:0005875 | BP | pattern specification process | 6.48E-11 |
| GO:0005975 | BP | multidimensional cell growth | 9.98E-11 |
| GO:0005982 | BP | starch metabolic process | 5.10E-10 |
| GO:0006006 | BP | mRNA modification | 7.69E-10 |
| GO:0006012 | BP | root hair elongation | 8.15E-10 |
| GO:0006032 | BP | NADH dehydrogenase complex (plastoquinone) assembly | 1.09E-09 |
| GO:0006066 | BP | cell proliferation | 1.52E-09 |
| GO:0006073 | BP | microtubule nucleation | 3.05E-09 |
| GO:0006098 | BP | raffinose catabolic process | 5.10E-09 |
| GO:0006226 | BP | glycine catabolic process | 6.97E-09 |
| GO:0006270 | BP | syncytium formation | 7.60E-09 |
| GO:0006275 | BP | regulation of protein dephosphorylation | 1.15E-08 |
| GO:0006334 | BP | auxin polar transport | 2.13E-08 |
| GO:0006364 | BP | coenzyme biosynthetic process | 8.02E-08 |
| GO:0006468 | BP | cysteine biosynthetic process | 1.10E-07 |
| GO:0006546 | BP | sulfur amino acid metabolic process | 1.18E-07 |
| GO:0006636 | BP | lipoate metabolic process | 2.07E-07 |
| GO:0006733 | BP | asymmetric cell division | 2.93E-07 |
| GO:0006766 | BP | histone phosphorylation | 3.03E-07 |
| GO:0006826 | BP | anther development | 3.07E-07 |
| GO:0006949 | BP | plastid translation | 3.24E-07 |
| GO:0006979 | BP | inositol catabolic process | 3.62E-07 |
| GO:0007018 | BP | unsaturated fatty acid biosynthetic process | 4.72E-07 |
| GO:0007020 | BP | galactose metabolic process | 6.61E-07 |
| GO:0007108 | BP | glucosinolate biosynthetic process | 1.23E-06 |
| GO:0007112 | BP | cell wall macromolecule catabolic process | 1.33E-06 |
| GO:0007169 | BP | aromatic amino acid family metabolic process | 1.43E-06 |
| GO:0007389 | BP | DNA replication initiation | 1.48E-06 |
| GO:0008061 | BP | root morphogenesis | 1.95E-06 |
| GO:0008152 | BP | anisotropic cell growth | 3.31E-06 |
| GO:0008266 | BP | regulation of cellular macromolecule biosynthetic process | 3.39E-06 |
| GO:0008283 | BP | regulation of DNA replication | 3.56E-06 |
| GO:0008356 | BP | detection of biotic stimulus | 3.77E-06 |
| GO:0008361 | BP | response to oxidative stress | 3.98E-06 |
| GO:0008447 | BP | somatic embryogenesis | 4.09E-06 |
| GO:0008652 | BP | stamen development | 4.40E-06 |
| GO:0008810 | BP | alcohol metabolic process | 5.21E-06 |
| GO:0008812 | BP | oxylipin biosynthetic process | 5.60E-06 |
| GO:0009044 | BP | iron ion transport | 5.63E-06 |
| GO:0009055 | BP | cytokinesis by cell plate formation | 6.45E-06 |
| GO:0009056 | BP | regulation of proton transport | 6.88E-06 |
| GO:0009072 | BP | vitamin metabolic process | 7.07E-06 |
| GO:0009106 | BP | histone H3-K9 methylation | 7.83E-06 |
| GO:0009108 | BP | glucose metabolic process | 8.57E-06 |
| GO:0009409 | BP | xylan catabolic process | 8.59E-06 |
| GO:0009414 | BP | oxidoreduction coenzyme metabolic process | 1.05E-05 |
| GO:0009416 | BP | positive gravitropism | 1.47E-05 |
| GO:0009505 | BP | chitin catabolic process | 1.64E-05 |
| GO:0009522 | BP | regulation of cell proliferation | 1.91E-05 |
| GO:0009523 | BP | response to cold | 2.06E-05 |
| GO:0009535 | BP | divalent metal ion transport | 2.86E-05 |
| GO:0009538 | BP | xyloglucan metabolic process | 3.53E-05 |
| GO:0009543 | BP | thylakoid membrane organization | 3.71E-05 |
| GO:0009544 | BP | cell differentiation | 3.79E-05 |
| GO:0009570 | BP | primary cell wall biogenesis | 3.80E-05 |
| GO:0009579 | BP | photosynthesis, light harvesting in photosystem I | 3.92E-05 |
| GO:0009595 | BP | regulation of G2/M transition of mitotic cell cycle | 3.95E-05 |
| GO:0009624 | BP | regulation of cyclin-dependent protein serine/threonine kinase activity | 4.41E-05 |
| GO:0009637 | BP | microsporocyte differentiation | 4.64E-05 |
| GO:0009654 | BP | pectin catabolic process | 4.82E-05 |
| GO:0009657 | BP | regulation of lipid metabolic process | 4.86E-05 |
| GO:0009664 | BP | cellular glucan metabolic process | 5.84E-05 |
| GO:0009697 | BP | carotenoid biosynthetic process | 6.02E-05 |
| GO:0009744 | BP | cellular amino acid biosynthetic process | 6.17E-05 |
| GO:0009765 | BP | response to nematode | 6.52E-05 |
| GO:0009768 | BP | monoterpenoid biosynthetic process | 7.04E-05 |
| GO:0009773 | BP | microtubule cytoskeleton organization | 0.000101204 |
| GO:0009814 | BP | metabolic process | 0.000111396 |
| GO:0009825 | BP | monocarboxylic acid metabolic process | 0.000117153 |
| GO:0009828 | BP | secondary metabolic process | 0.00012036 |
| GO:0009832 | BP | zinc ion homeostasis | 0.000123988 |
| GO:0009833 | BP | salicylic acid biosynthetic process | 0.000133325 |
| GO:0009926 | BP | shikimate metabolic process | 0.00013623 |
| GO:0009932 | BP | lignin catabolic process | 0.000140458 |
| GO:0009941 | BP | nucleosome assembly | 0.000144027 |
| GO:0009958 | BP | cell wall modification | 0.00016584 |
| GO:0009971 | BP | spindle assembly | 0.000167445 |
| GO:0010007 | BP | cellular cation homeostasis | 0.00017425 |
| GO:0010015 | BP | detection of mechanical stimulus | 0.000178747 |
| GO:0010027 | BP | barbed-end actin filament capping | 0.000207351 |
| GO:0010075 | BP | protein phosphorylation | 0.000211209 |
| GO:0010103 | BP | acropetal auxin transport | 0.000223682 |
| GO:0010106 | BP | protein polymerization | 0.000237243 |
| GO:0010114 | BP | regulation of multi-organism process | 0.000239117 |
| GO:0010155 | BP | nitrate transport | 0.000287286 |
| GO:0010207 | BP | cell wall modification involved in multidimensional cell growth | 0.000297965 |
| GO:0010218 | BP | anastral spindle assembly involved in male meiosis | 0.000309144 |
| GO:0010242 | BP | very long-chain fatty acid metabolic process | 0.00033526 |
| GO:0010245 | BP | single-organism metabolic process | 0.000363245 |
| GO:0010258 | BP | response to light stimulus | 0.000433562 |
| GO:0010262 | BP | carpel development | 0.000437425 |
| GO:0010264 | BP | plant-type cell wall loosening | 0.000438115 |
| GO:0010287 | BP | dUMP biosynthetic process | 0.000455187 |
| GO:0010319 | BP | dUTP metabolic process | 0.000455187 |
| GO:0010329 | BP | radial microtubular system formation | 0.000459139 |
| GO:0010389 | BP | response to sucrose stimulus | 0.000466049 |
| GO:0010411 | BP | sepal formation | 0.000501248 |
| GO:0010480 | BP | respiratory burst involved in defense response | 0.000511994 |
| GO:0010541 | BP | defense response, incompatible interaction | 0.000601301 |
| GO:0010583 | BP | catabolic process | 0.000605797 |
| GO:0010598 | BP | response to herbivore | 0.000636107 |
| GO:0010817 | BP | defense response to bacterium | 0.000683617 |
| GO:0015238 | BP | myo-inositol hexakisphosphate biosynthetic process | 0.000772734 |
| GO:0015297 | BP | cytokinesis, initiation of separation | 0.000776296 |
| GO:0015706 | BP | response to cyclopentenone | 0.000789706 |
| GO:0015979 | BP | glucosinolate metabolic process | 0.000800203 |
| GO:0015995 | BP | cellular response to iron ion starvation | 0.000806695 |
| GO:0016020 | BP | cellular response to chitin | 0.000830381 |
| GO:0016021 | BP | mucilage metabolic process involved in seed coat development | 0.000840424 |
| GO:0016023 | BP | raffinose family oligosaccharide biosynthetic process | 0.000871801 |
| GO:0016099 | BP | petal formation | 0.000954551 |
| GO:0016117 | BP | response to water deprivation | 0.000985235 |
| GO:0016161 | CC | chloroplast thylakoid membrane | 2.47E-58 |
| GO:0016491 | CC | extracellular region | 4.09E-29 |
| GO:0016556 | CC | apoplast | 5.94E-27 |
| GO:0016572 | CC | plastoglobule | 2.50E-21 |
| GO:0016614 | CC | plant-type cell wall | 4.09E-17 |
| GO:0016630 | CC | chloroplast stroma | 2.50E-14 |
| GO:0016655 | CC | chloroplast envelope | 4.23E-14 |
| GO:0016709 | CC | NAD(P)H dehydrogenase complex (plastoquinone) | 7.95E-14 |
| GO:0016760 | CC | integral to membrane | 1.48E-13 |
| GO:0016762 | CC | chloroplast thylakoid lumen | 5.70E-13 |
| GO:0016851 | CC | photosystem I | 1.24E-11 |
| GO:0016998 | CC | microtubule associated complex | 2.23E-11 |
| GO:0019216 | CC | photosystem I reaction center | 9.98E-11 |
| GO:0019252 | CC | microtubule | 1.97E-10 |
| GO:0019288 | CC | stromule | 8.46E-10 |
| GO:0019310 | CC | cytoplasmic membrane-bounded vesicle | 7.15E-09 |
| GO:0019344 | CC | oxygen evolving complex | 9.46E-09 |
| GO:0019632 | CC | anchored to plasma membrane | 1.26E-07 |
| GO:0019684 | CC | cell wall | 1.11E-06 |
| GO:0019748 | CC | thylakoid | 1.22E-06 |
| GO:0019760 | CC | extrinsic to membrane | 1.24E-06 |
| GO:0019761 | CC | photosystem II | 1.98E-06 |
| GO:0019825 | CC | plant extracellular matrix | 4.35E-06 |
| GO:0019898 | CC | membrane | 7.70E-06 |
| GO:0020037 | CC | thylakoid lumen | 3.37E-05 |
| GO:0030003 | CC | chloroplast ATP synthase complex | 5.81E-05 |
| GO:0030076 | CC | nucleosome | 7.42E-05 |
| GO:0030093 | CC | anchored to membrane | 0.000248777 |
| GO:0030095 | CC | chloroplast photosystem II | 0.000507439 |
| GO:0030154 | CC | light-harvesting complex | 0.00072772 |
| GO:0030570 | CC | chloroplast photosystem I | 0.000805219 |
| GO:0030599 | CC | magnesium chelatase complex | 0.000931048 |
| GO:0031225 | MF | inositol 3-alpha-galactosyltransferase activity | 4.66E-12 |
| GO:0031408 | MF | NADP binding | 7.14E-11 |
| GO:0031977 | MF | NADPH dehydrogenase activity | 3.57E-10 |
| GO:0032440 | MF | galactinol-sucrose galactosyltransferase activity | 5.18E-10 |
| GO:0032544 | MF | hydrolase activity, hydrolyzing O-glycosyl compounds | 8.25E-10 |
| GO:0032787 | MF | glyceraldehyde-3-phosphate dehydrogenase (NADP+) (phosphorylating) activity | 2.96E-09 |
| GO:0034484 | MF | pectinesterase activity | 8.40E-09 |
| GO:0035251 | MF | metal ion binding | 1.48E-08 |
| GO:0035304 | MF | chitin binding | 4.49E-08 |
| GO:0042127 | MF | oxidoreductase activity, acting on NAD(P)H, quinone or similar compound as acceptor | 4.75E-08 |
| GO:0042545 | MF | identical protein binding | 1.07E-07 |
| GO:0042547 | MF | microtubule motor activity | 3.37E-07 |
| GO:0042742 | MF | poly(U) RNA binding | 3.62E-07 |
| GO:0042802 | MF | inositol oxygenase activity | 3.62E-07 |
| GO:0042973 | MF | protochlorophyllide reductase activity | 4.99E-07 |
| GO:0043085 | MF | heme binding | 1.04E-06 |
| GO:0043086 | MF | cellulase activity | 1.05E-06 |
| GO:0043169 | MF | cation binding | 1.13E-06 |
| GO:0043481 | MF | 2-alkenal reductase [NAD(P)] activity | 1.33E-06 |
| GO:0043900 | MF | aspartyl esterase activity | 3.17E-06 |
| GO:0044710 | MF | oxidoreductase activity, acting on paired donors, with incorporation or reduction of molecular oxygen, NAD(P)H as one donor, and incorporation of one atom of oxygen | 3.67E-06 |
| GO:0045330 | MF | ferric-chelate reductase activity | 4.52E-06 |
| GO:0045490 | MF | 3-dehydroquinate dehydratase activity | 4.90E-06 |
| GO:0045493 | MF | shikimate 3-dehydrogenase (NADP+) activity | 4.90E-06 |
| GO:0046080 | MF | UDP-glucosyltransferase activity | 6.54E-06 |
| GO:0046274 | MF | carboxypeptidase activity | 8.44E-06 |
| GO:0046556 | MF | pectate lyase activity | 9.54E-06 |
| GO:0046577 | MF | serine-type endopeptidase activity | 9.82E-06 |
| GO:0046658 | MF | enzyme inhibitor activity | 1.06E-05 |
| GO:0046872 | MF | cellulose synthase (UDP-forming) activity | 1.21E-05 |
| GO:0047100 | MF | electron carrier activity | 1.43E-05 |
| GO:0047213 | MF | iron ion binding | 1.58E-05 |
| GO:0047216 | MF | raffinose alpha-galactosidase activity | 1.67E-05 |
| GO:0047268 | MF | polygalacturonase activity | 1.84E-05 |
| GO:0047274 | MF | beta-galactosidase activity | 1.89E-05 |
| GO:0047364 | MF | L-ascorbate oxidase activity | 1.95E-05 |
| GO:0047958 | MF | Rho guanyl-nucleotide exchange factor activity | 2.12E-05 |
| GO:0048046 | MF | aminomethyltransferase activity | 2.24E-05 |
| GO:0048196 | MF | oxygen evolving activity | 3.79E-05 |
| GO:0048359 | MF | glycine dehydrogenase (decarboxylating) activity | 5.56E-05 |
| GO:0048440 | MF | beta-amylase activity | 6.15E-05 |
| GO:0048443 | MF | desulfoglucosinolate sulfotransferase activity | 6.99E-05 |
| GO:0048451 | MF | galactinol-raffinose galactosyltransferase activity | 7.17E-05 |
| GO:0048453 | MF | chitinase activity | 7.75E-05 |
| GO:0048653 | MF | xyloglucan:xyloglucosyl transferase activity | 7.85E-05 |
| GO:0048767 | MF | glycine:2-oxoglutarate aminotransferase activity | 8.77E-05 |
| GO:0050113 | MF | hydroquinone:oxygen oxidoreductase activity | 0.000116371 |
| GO:0050321 | MF | polyneuridine-aldehyde esterase activity | 0.000153395 |
| GO:0050529 | MF | alpha-N-arabinofuranosidase activity | 0.00015454 |
| GO:0050661 | MF | glucan endo-1,3-beta-D-glucosidase activity | 0.000160029 |
| GO:0050982 | MF | inward rectifier potassium channel activity | 0.000164502 |
| GO:0051016 | MF | antiporter activity | 0.00017103 |
| GO:0051211 | MF | L-alanine:2-oxoglutarate aminotransferase activity | 0.000229178 |
| GO:0051225 | MF | oxidoreductase activity | 0.000263814 |
| GO:0051258 | MF | xylan 1,4-beta-xylosidase activity | 0.000287388 |
| GO:0051567 | MF | auxin efflux transmembrane transporter activity | 0.000316855 |
| GO:0052692 | MF | anthocyanidin 3-O-glucosyltransferase activity | 0.000352077 |
| GO:0052716 | MF | tau-protein kinase activity | 0.000386594 |
| GO:0055069 | MF | dUTP diphosphatase activity | 0.000455187 |
| GO:0055114 | MF | choline dehydrogenase activity | 0.000580718 |
| GO:0070838 | MF | oxygen binding | 0.000607096 |
| GO:0071323 | MF | oxidoreductase activity, acting on CH-OH group of donors | 0.00066622 |
| GO:0080027 | MF | drug transmembrane transporter activity | 0.000683325 |
| GO:0080167 | MF | long-chain-alcohol oxidase activity | 0.000788615 |
| GO:2000112 | MF | magnesium chelatase activity | 0.000931048 |
