# Supplementary material for: De Novo Sequencing and Comparative Analysis of Schima superba Seedlings to Explore the Response to Drought Stress
Source: PLoS One. 2016 Dec 8;11(12):e0166975. doi: 10.1371/journal.pone.0166975 (PMC5145176; doi:10.1371/journal.pone.0166975)
Supplement: S3 Table — (DOCX) [file pone.0166975.s003.docx]

**S3 Table. GO enrichment of down-regulated DEGs in the drought stress treatment.**

| **GOID** | **Ontology** | **Terms** | **P value** |
| --- | --- | --- | --- |
| GO:0003723 | BP | cysteine biosynthetic process | 0 |
| GO:0005730 | BP | glucosinolate biosynthetic process | 0 |
| GO:0005829 | BP | asymmetric cell division | 0 |
| GO:0080008 | BP | auxin polar transport | 9.21E-10 |
| GO:0004842 | BP | regulation of protein dephosphorylation | 1.25E-09 |
| GO:0001510 | BP | coenzyme biosynthetic process | 5.46E-09 |
| GO:0005515 | BP | raffinose catabolic process | 1.75E-08 |
| GO:0006486 | BP | syncytium formation | 2.05E-08 |
| GO:0016567 | BP | glycine catabolic process | 2.33E-08 |
| GO:0006626 | BP | histone phosphorylation | 9.58E-08 |
| GO:0033044 | BP | galactose metabolic process | 1.28E-07 |
| GO:0006396 | BP | cell proliferation | 2.90E-07 |
| GO:0006606 | BP | microtubule nucleation | 4.25E-07 |
| GO:0008026 | BP | NADH dehydrogenase complex (plastoquinone) assembly | 5.46E-07 |
| GO:0006635 | BP | root hair elongation | 1.08E-06 |
| GO:0016579 | BP | unsaturated fatty acid biosynthetic process | 2.11E-06 |
| GO:0005802 | BP | mRNA modification | 2.31E-06 |
| GO:0005488 | BP | rRNA processing | 2.01E-05 |
| GO:0000398 | BP | multidimensional cell growth | 2.17E-05 |
| GO:0006355 | BP | regulation of hormone levels | 2.28E-05 |
| GO:0097159 | BP | maltose metabolic process | 3.10E-05 |
| GO:1901363 | BP | pattern specification process | 3.10E-05 |
| GO:0006094 | BP | polysaccharide catabolic process | 3.22E-05 |
| GO:0006499 | BP | response to karrikin | 3.51E-05 |
| GO:0000166 | BP | microtubule-based movement | 3.55E-05 |
| GO:0042138 | BP | starch metabolic process | 4.43E-05 |
| GO:0016571 | BP | inositol catabolic process | 6.59E-05 |
| GO:0006412 | BP | starch biosynthetic process | 6.83E-05 |
| GO:0005525 | BP | plastid organization | 8.43E-05 |
| GO:0006457 | BP | cell tip growth | 0.000116468 |
| GO:0006413 | BP | photosynthesis, light reaction | 0.000120317 |
| GO:0009630 | BP | plant-type cell wall biogenesis | 0.00014525 |
| GO:0005768 | BP | photosystem II assembly | 0.000149308 |
| GO:0008234 | BP | photosynthetic electron transport in photosystem I | 0.000167746 |
| GO:0003743 | BP | plant-type cell wall organization | 0.000191429 |
| GO:0016926 | BP | carbohydrate metabolic process | 0.000201153 |
| GO:0006888 | BP | isopentenyl diphosphate biosynthetic process, mevalonate-independent pathway | 0.000203543 |
| GO:0005643 | BP | lipoate metabolic process | 0.000214341 |
| GO:0003735 | BP | response to blue light | 0.000235155 |
| GO:0004402 | BP | sulfur amino acid metabolic process | 0.000243829 |
| GO:0005737 | BP | response to red light | 0.000316301 |
| GO:0009910 | BP | positive regulation of catalytic activity | 0.000323133 |
| GO:0009507 | BP | transmembrane receptor protein tyrosine kinase signaling pathway | 0.000343037 |
| GO:0009888 | BP | plastid translation | 0.000352702 |
| GO:0009640 | BP | photosynthesis | 0.000382568 |
| GO:0000902 | BP | polysaccharide biosynthetic process | 0.00040719 |
| GO:0006007 | BP | regulation of cell size | 0.000414645 |
| GO:0004712 | BP | stomatal complex morphogenesis | 0.000462151 |
| GO:0008219 | BP | anther development | 0.000531617 |
| GO:0004683 | BP | negative regulation of catalytic activity | 0.000534937 |
| GO:0003899 | BP | pentose-phosphate shunt | 0.000588327 |
| GO:0051788 | BP | oxidation-reduction process | 0.000643456 |
| GO:0043687 | BP | regulation of meristem growth | 0.000670971 |
| GO:0042393 | BP | response to far red light | 0.00069448 |
| GO:0035091 | BP | chlorophyll biosynthetic process | 0.000757013 |
| GO:0035196 | BP | photosynthesis, light harvesting | 0.000866398 |
| GO:0006366 | BP | anthocyanin accumulation in tissues in response to UV light | 0.000874016 |
| GO:0005634 | CC | anchored to plasma membrane | 0 |
| GO:0005739 | CC | cell wall | 0 |
| GO:0006511 | CC | thylakoid | 0 |
| GO:0006623 | CC | oxygen evolving complex | 6.38E-09 |
| GO:0048573 | CC | cytoplasmic membrane-bounded vesicle | 5.04E-08 |
| GO:0045132 | CC | stromule | 1.30E-06 |
| GO:0005794 | CC | photosystem I reaction center | 7.15E-06 |
| GO:0006406 | CC | microtubule | 5.20E-05 |
| GO:0016558 | CC | microtubule associated complex | 5.64E-05 |
| GO:0007062 | CC | chloroplast thylakoid lumen | 0.000100623 |
| GO:0006944 | CC | plastoglobule | 0.000234015 |
| GO:0080129 | CC | chloroplast stroma | 0.00024431 |
| GO:0008284 | CC | chloroplast envelope | 0.000260215 |
| GO:0004222 | CC | photosystem I | 0.00026047 |
| GO:0022625 | CC | plant-type cell wall | 0.000349114 |
| GO:0008568 | CC | integral to membrane | 0.00040171 |
| GO:0004386 | CC | NAD(P)H dehydrogenase complex (plastoquinone) | 0.000436664 |
| GO:0050665 | CC | apoplast | 0.000822191 |
| GO:0048366 | CC | extracellular region | 0.000862082 |
| GO:0016036 | CC | chloroplast thylakoid membrane | 0.000903469 |
| GO:0003676 | MF | identical protein binding | 0 |
| GO:0008270 | MF | cation binding | 0 |
| GO:0000956 | MF | oxidoreductase activity, acting on NAD(P)H, quinone or similar compound as acceptor | 2.53E-11 |
| GO:0016192 | MF | chitin binding | 1.72E-10 |
| GO:0048193 | MF | metal ion binding | 1.36E-09 |
| GO:0010388 | MF | microtubule motor activity | 1.18E-08 |
| GO:0009560 | MF | pectinesterase activity | 1.80E-08 |
| GO:0009220 | MF | glyceraldehyde-3-phosphate dehydrogenase (NADP+) (phosphorylating) activity | 5.33E-07 |
| GO:0006487 | MF | hydrolase activity, hydrolyzing O-glycosyl compounds | 1.13E-06 |
| GO:0006886 | MF | galactinol-sucrose galactosyltransferase activity | 1.21E-05 |
| GO:0030276 | MF | protochlorophyllide reductase activity | 1.58E-05 |
| GO:0045892 | MF | NADPH dehydrogenase activity | 1.73E-05 |
| GO:0007131 | MF | NADP binding | 3.42E-05 |
| GO:0051604 | MF | cellulase activity | 4.71E-05 |
| GO:0010638 | MF | poly(U) RNA binding | 0.000106112 |
| GO:0048589 | MF | heme binding | 0.000166384 |
| GO:0000151 | MF | inositol 3-alpha-galactosyltransferase activity | 0.000302407 |
| GO:0016573 | MF | inositol oxygenase activity | 0.000445864 |
