# Supplementary material for: De Novo Sequencing and Comparative Analysis of Schima superba Seedlings to Explore the Response to Drought Stress
Source: PLoS One. 2016 Dec 8;11(12):e0166975. doi: 10.1371/journal.pone.0166975 (PMC5145176; doi:10.1371/journal.pone.0166975)
Supplement: S4 Table — (DOCX) [file pone.0166975.s004.docx]

**S4 Table. KEGG pathway enrichment of up-regulated DEGs in the drought treatment.**

| Pathway | Sample NO. | Pvalue |
| --- | --- | --- |
| Photosynthesis - antenna proteins | 41 | 1.81E-30 |
| Photosynthesis | 67 | 9.96E-27 |
| Metabolic pathways | 1147 | 1.01E-25 |
| Biosynthesis of secondary metabolites | 604 | 2.12E-24 |
| Phenylpropanoid biosynthesis | 141 | 6.81E-18 |
| Starch and sucrose metabolism | 180 | 3.82E-17 |
| Flavone and flavonol biosynthesis | 70 | 3.11E-16 |
| Zeatin biosynthesis | 68 | 8.58E-12 |
| Pentose and glucuronate interconversions | 96 | 2.28E-11 |
| Stilbenoid, diarylheptanoid and gingerol biosynthesis | 80 | 4.24E-11 |
| Diterpenoid biosynthesis | 45 | 5.34E-11 |
| Carbon fixation in photosynthetic organisms | 63 | 1.47E-10 |
| Flavonoid biosynthesis | 89 | 2.59E-10 |
| Ascorbate and aldarate metabolism | 64 | 7.22E-10 |
| Plant hormone signal transduction | 365 | 2.28E-09 |
| Cutin, suberine and wax biosynthesis | 49 | 6.24E-09 |
| Galactose metabolism | 54 | 1.32E-08 |
| Glucosinolate biosynthesis | 21 | 3.27E-08 |
| Cyanoamino acid metabolism | 53 | 3.31E-07 |
| Limonene and pinene degradation | 54 | 5.63E-07 |
| Tryptophan metabolism | 35 | 7.76E-07 |
| Other glycan degradation | 43 | 8.20E-07 |
| Phenylalanine metabolism | 56 | 4.63E-06 |
| Carotenoid biosynthesis | 47 | 6.35E-06 |
| Glycosphingolipid biosynthesis - ganglio series | 18 | 1.42E-05 |
| Ether lipid metabolism | 165 | 1.72E-05 |
| Glycerophospholipid metabolism | 196 | 2.72E-05 |
| Indole alkaloid biosynthesis | 13 | 4.89E-05 |
| Glycosaminoglycan degradation | 19 | 0.000288723 |
| Sesquiterpenoid and triterpenoid biosynthesis | 14 | 0.000283941 |
| Isoflavonoid biosynthesis | 22 | 0.000344974 |
| Ubiquinone and other terpenoid-quinone biosynthesis | 27 | 0.000471823 |
| Linoleic acid metabolism | 16 | 0.000582179 |
| Cysteine and methionine metabolism | 53 | 0.000709017 |
| Isoquinoline alkaloid biosynthesis | 20 | 0.000820015 |
