# Supplementary material for: De Novo Sequencing and Comparative Analysis of Schima superba Seedlings to Explore the Response to Drought Stress
Source: PLoS One. 2016 Dec 8;11(12):e0166975. doi: 10.1371/journal.pone.0166975 (PMC5145176; doi:10.1371/journal.pone.0166975)
Supplement: S5 Table — (DOCX) [file pone.0166975.s005.docx]

**S5 Table. KEGG pathway enrichment of down-regulated DEGs in the drought treatment.**

| Pathway | Sample NO. | Pvalue |
| --- | --- | --- |
| Spliceosome | 51 | 0 |
| RNA transport | 93 | 0 |
| Protein processing in endoplasmic reticulum | 51 | 0 |
| mRNA surveillance pathway | 27 | 0 |
| Ribosome biogenesis in eukaryotes | 25 | 0 |
| Ubiquitin mediated proteolysis | 38 | 5.06E-10 |
| Ribosome | 33 | 2.95E-09 |
| N-Glycan biosynthesis | 1 | 3.86E-09 |
| RNA degradation | 36 | 7.50E-09 |
| Purine metabolism | 62 | 1.02E-07 |
| Basal transcription factors | 7 | 1.13E-07 |
| Nucleotide excision repair | 9 | 1.06E-06 |
| RNA polymerase | 33 | 4.07E-06 |
| Pyrimidine metabolism | 60 | 6.60E-06 |
| Homologous recombination | 3 | 7.55E-06 |
| Phosphatidylinositol signaling system | 16 | 2.41E-05 |
| Mismatch repair | 4 | 0.000201536 |
| Pantothenate and CoA biosynthesis | 1 | 0.0002101 |
| Citrate cycle (TCA cycle) | 6 | 0.000279228 |
| Circadian rhythm - plant | 31 | 0.000363237 |
| Proteasome | 6 | 0.000628223 |
| Base excision repair | 10 | 0.000694182 |
| Protein export | 5 | 0.000797544 |
