# Supplementary material for: De Novo Sequencing and Comparative Analysis of Schima superba Seedlings to Explore the Response to Drought Stress
Source: PLoS One. 2016 Dec 8;11(12):e0166975. doi: 10.1371/journal.pone.0166975 (PMC5145176; doi:10.1371/journal.pone.0166975)
Supplement: S6 Table — (DOCX) [file pone.0166975.s006.docx]

S6 Table. The stomatal conductance (GS) data measured from the first six days (unit: mmol・m-2・s-1)

| Replicates | Treatments | GS in the 1st day | | GS in the 2st day | | GS in the 3st day | | GS in the 4st day | | GS in the 5st day | | GS in the 6st day | |
| --- | --- | --- | --- | --- | --- | --- | --- | --- | --- | --- | --- | --- | --- |
|  |  | Mean | SD | Mean | SD | Mean | SD | Mean | SD | Mean | SD | Mean | SD |
| 1 | DT | 0.04753 | 0.00042 | 0.04130 | 0.00056 | 0.04833 | 0.00059 | 0.05577 | 0.00051 | 0.04080 | 0.00053 | 0.01177 | 0.00091 |
| 1 | DT | 0.17433 | 0.00081 | 0.17987 | 0.00075 | 0.19167 | 0.00115 | 0.18330 | 0.00070 | 0.15983 | 0.00090 | 0.13073 | 0.00057 |
| 1 | DT | 0.09247 | 0.00015 | 0.10127 | 0.00055 | 0.09916 | 0.00084 | 0.10747 | 0.00095 | 0.08583 | 0.00025 | 0.06140 | 0.00046 |
| 1 | DT | 0.03963 | 0.00021 | 0.04673 | 0.00006 | 0.04583 | 0.00025 | 0.05603 | 0.00060 | 0.03513 | 0.00040 | 0.01057 | 0.00068 |
| 1 | DT | 0.07633 | 0.00276 | 0.08710 | 0.00040 | 0.09217 | 0.00032 | 0.08537 | 0.00045 | 0.07287 | 0.00085 | 0.04893 | 0.00051 |
| 1 | DT | 0.10470 | 0.00110 | 0.10858 | 0.00003 | 0.10263 | 0.00045 | 0.10443 | 0.00047 | 0.09303 | 0.00100 | 0.06390 | 0.00020 |
| 1 | CK | 0.08330 | 0.00105 | 0.09467 | 0.00134 | 0.09310 | 0.00062 | 0.09320 | 0.00040 | 0.11643 | 0.00070 | 0.12480 | 0.00070 |
| 1 | CK | 0.05843 | 0.00015 | 0.06507 | 0.00087 | 0.07213 | 0.00205 | 0.06743 | 0.00053 | 0.06930 | 0.00046 | 0.06787 | 0.00060 |
| 1 | CK | 0.04853 | 0.00012 | 0.05167 | 0.00045 | 0.05920 | 0.00062 | 0.05493 | 0.00067 | 0.05843 | 0.00025 | 0.05503 | 0.00015 |
| 1 | CK | 0.07080 | 0.00040 | 0.08153 | 0.00038 | 0.0996 | 0.00025 | 0.09500 | 0.00036 | 0.09327 | 0.00042 | 0.09253 | 0.00036 |
| 1 | CK | 0.13567 | 0.00115 | 0.13500 | 0.00361 | 0.13233 | 0.00351 | 0.12533 | 0.00058 | 0.13060 | 0.00069 | 0.12800 | 0.00557 |
| 1 | CK | 0.11167 | 0.00115 | 0.12133 | 0.00058 | 0.11733 | 0.00058 | 0.13367 | 0.00058 | 0.12633 | 0.00058 | 0.13467 | 0.00058 |
| 2 | DT | 0.04603 | 0.00085 | 0.05323 | 0.00042 | 0.06717 | 0.00031 | 0.06313 | 0.00031 | 0.04810 | 0.00167 | 0.02220 | 0.00066 |
| 2 | DT | 0.16093 | 0.00115 | 0.16827 | 0.00050 | 0.17730 | 0.00044 | 0.17333 | 0.00211 | 0.15877 | 0.00061 | 0.13170 | 0.00053 |
| 2 | DT | 0.09060 | 0.00105 | 0.09930 | 0.00056 | 0.10053 | 0.00061 | 0.10520 | 0.00044 | 0.08633 | 0.00050 | 0.05033 | 0.00064 |
| 2 | DT | 0.02690 | 0.00020 | 0.03050 | 0.00185 | 0.03347 | 0.00015 | 0.03627 | 0.00060 | 0.02070 | 0.00056 | 0.00860 | 0.00056 |
| 2 | DT | 0.07553 | 0.00006 | 0.08250 | 0.00082 | 0.09157 | 0.00023 | 0.08930 | 0.00391 | 0.06933 | 0.00045 | 0.03133 | 0.00021 |
| 2 | DT | 0.10303 | 0.00068 | 0.09907 | 0.00025 | 0.10357 | 0.00042 | 0.10030 | 0.00046 | 0.08600 | 0.00056 | 0.05107 | 0.00071 |
| 2 | CK | 0.07827 | 0.00047 | 0.08723 | 0.00040 | 0.09597 | 0.00059 | 0.09277 | 0.00040 | 0.10113 | 0.00154 | 0.10403 | 0.00064 |
| 2 | CK | 0.05150 | 0.00030 | 0.05850 | 0.00036 | 0.06330 | 0.00056 | 0.06860 | 0.00010 | 0.06313 | 0.00031 | 0.06563 | 0.00038 |
| 2 | CK | 0.04573 | 0.00278 | 0.05307 | 0.00061 | 0.06647 | 0.00059 | 0.06777 | 0.00045 | 0.06107 | 0.00071 | 0.06203 | 0.00049 |
| 2 | CK | 0.06943 | 0.00045 | 0.07610 | 0.00026 | 0.07270 | 0.00053 | 0.07827 | 0.00032 | 0.08250 | 0.00036 | 0.07757 | 0.00029 |
| 2 | CK | 0.13467 | 0.00058 | 0.14080 | 0.00036 | 0.14853 | 0.00040 | 0.15367 | 0.00058 | 0.15767 | 0.00058 | 0.15510 | 0.00036 |
| 2 | CK | 0.10670 | 0.00020 | 0.09683 | 0.00012 | 0.15233 | 0.00058 | 0.12500 | 0.00100 | 0.12617 | 0.00025 | 0.12357 | 0.00040 |
| 3 | DT | 0.04260 | 0.00089 | 0.05100 | 0.00036 | 0.05837 | 0.00038 | 0.05340 | 0.00035 | 0.03780 | 0.00036 | 0.01213 | 0.00040 |
| 3 | DT | 0.11247 | 0.00351 | 0.09907 | 0.00061 | 0.10400 | 0.00262 | 0.09977 | 0.00081 | 0.08523 | 0.00074 | 0.05920 | 0.00062 |
| 3 | DT | 0.07897 | 0.00083 | 0.07503 | 0.00006 | 0.08113 | 0.00029 | 0.07773 | 0.00015 | 0.06537 | 0.00055 | 0.04010 | 0.00066 |
| 3 | DT | 0.02483 | 0.00031 | 0.02583 | 0.00080 | 0.03190 | 0.00061 | 0.03237 | 0.00006 | 0.02163 | 0.00031 | 0.00430 | 0.00035 |
| 3 | DT | 0.04973 | 0.00076 | 0.05210 | 0.00060 | 0.05777 | 0.00060 | 0.05647 | 0.00040 | 0.04163 | 0.00060 | 0.01460 | 0.00040 |
| 3 | DT | 0.10237 | 0.00140 | 0.10133 | 0.00040 | 0.10017 | 0.00035 | 0.10970 | 0.00056 | 0.09210 | 0.00036 | 0.06503 | 0.00021 |
| 3 | CK | 0.07600 | 0.00050 | 0.07210 | 0.00053 | 0.06197 | 0.00021 | 0.07973 | 0.00047 | 0.09200 | 0.00010 | 0.09773 | 0.00031 |
| 3 | CK | 0.04910 | 0.00010 | 0.04917 | 0.00042 | 0.05723 | 0.00032 | 0.06863 | 0.00025 | 0.06347 | 0.00012 | 0.06060 | 0.00010 |
| 3 | CK | 0.02773 | 0.00006 | 0.03013 | 0.00031 | 0.03097 | 0.00006 | 0.03900 | 0.00036 | 0.03830 | 0.00035 | 0.03517 | 0.00006 |
| 3 | CK | 0.06750 | 0.00052 | 0.09330 | 0.00075 | 0.08907 | 0.00047 | 0.10113 | 0.00006 | 0.10120 | 0.00066 | 0.11337 | 0.00084 |
| 3 | CK | 0.11933 | 0.00058 | 0.11447 | 0.00067 | 0.12967 | 0.00115 | 0.11910 | 0.00046 | 0.11233 | 0.00058 | 0.11250 | 0.00036 |
| 3 | CK | 0.08830 | 0.00026 | 0.10700 | 0.00026 | 0.09440 | 0.00017 | 0.09250 | 0.00056 | 0.10400 | 0.00046 | 0.10150 | 0.00017 |

There were 3 repeats in every measure of every day. The mean value of every measure was used in the analysis.
