# Supplementary material for: De Novo Sequencing and Comparative Analysis of Schima superba Seedlings to Explore the Response to Drought Stress
Source: PLoS One. 2016 Dec 8;11(12):e0166975. doi: 10.1371/journal.pone.0166975 (PMC5145176; doi:10.1371/journal.pone.0166975)
Supplement: S7 Table — (DOCX) [file pone.0166975.s007.docx]

**S7 Table.** **Primers for qRT-PCR analysis.**

| Unigene No. | **Primer set** |  |
| --- | --- | --- |
|  | **Forward primer (5’-3’)** | **Reverse primer (5’-3’)** |
| CL9206.Contig | GCCAAGCATCTTGTGGCAAG | TCCAAATCCAGCCCTCCATT |
| CL9904.Contig1 | TGGATAGGCACCTTGGACCA | GAGGCGGTCAATGATGGAGA |
| Unigene12027 | ACCCTGCTCAGCAGAATTCC | AGCCATCCTTCCACCGTATG |
| Unigene17199 | AGCTGAGTTTCCCGAGGACT | TCCGGCTTTAGTACAGCTGG |
| Unigene18478 | GTCGTTGTTTGCAATTCCGC | GGCCATTGCAGAACTCCAGA |
| Unigene18823 | GCGACGATGAGATCAATGCC | TCAGCTCCTCCAATCCACCA |
| Unigene28348 | TCCTAGGAATGGCAACAGGC | TGATTTCCCGATCCCCTTCT |
| Unigene3045 | CCCTTTTGCTGAGCAGGACT | CCCGCAGCACAGTAGTGAAC |
| Unigene6031 | AGACGGCGAAGCTGAAACAG | TTCTGCGACCCGAAATCATT |
| Unigene6898 | AAGCCCAGAACTGCCAGGAT | TGTTTGGTTCAAAGCCGGAG |
| CL112.Contig1 | GCCAGAGTCTCCGAGCCAAC | ATCGCGGTGGAGACGATTTC |
| CL1362.Contig2 | GCATTGGCACAACTCCATCC | ATCAAAGCTGCGGTTACCCC |
| CL1804.Contig1 | TAAACTCTGACGGCGCGATC | CCTCACTGTCCTCACCGACG |
| CL2291.Contig3 | ATCCTCTTCCCTCCGTTCCA | CCAGCTCCAGTAGACACCGG |
| CL2363.Contig2 | GAAGGTGGTCGATGCATTTG | ACCACCGCCATCGTACACTA |
| CL3065.Contig3 | GCATGATGAGCACAAGACCC | TGCAGTGCTACCACATGCTG |
| CL4150.Contig2 | GCGTTTTGGTCGTGCTTTTC | CCAAACTCGCCGATCAGAAC |
| CL5523.Contig3 | GCATTCCGGTATCTTTCCGG | ATCCTCTGCCGAAAACAGCC |
| 18s | GGAGTATGGTCGCAAGGCTG | GGACCTGGTAAGTTTCCCCG |
